# PB.32: Does vacuum-assisted biopsy decrease the B3 rate in stereotactic biopsy of breast lesions?

**DOI:** 10.1186/bcr3532

**Published:** 2013-11-08

**Authors:** T Seaton, S Khan, V Stewart, G Ralliegh, N Zaman, N Barrett, S Comitis, A Gupta, W Svensson, A Lim, R Williamson, D Cunningham

**Affiliations:** 1 Charing Cross Hospital, West London Breast Screening Service, London, UK

## Introduction

Vacuum-assisted biopsy (VAB) systems have been shown to outperform 14G core needle biopsy (CNB) reducing the need for diagnostic or multiple surgeries. We introduced VAB in 2011 with the aim of reducing our B3 rate and increasing rate of preoperative diagnosis of invasive cancer.

## Methods

Mammographic abnormalities requiring stereotactic biopsy in a defined period which had either 14G CNB or VAB were included. Data collated included mammographic findings, biopsy and surgical outcome.

## Results

Ninety 14G-CNBs (November 2010 to May 2011) were compared with 78 VABS (July 2012 to January 2013). There was no reduction in our B3 rate (12 and 13 cases respectively) although VAB had higher sensitivity with better correlation between biopsy diagnosis and surgical histology (77% compared with 42%). Four of 12 (25%) B3 lesions diagnosed following 14G CNB were upgraded to DCIS following surgical excision compared with no cases following VAB. Preoperative invasive malignancy diagnostic rates were unchanged, 15% of cases were upgraded in both groups.

## Conclusion

VAB has not reduced our B3 rate but has increased the accuracy of our preoperative diagnosis of non-invasive cancer supporting current theories that patients who have undergone vacuum-assisted biopsy may not require surgical diagnostic excision. However, we recognise the number of cases is low and further cases will be analysed and presented.

